# A qualitative evaluation of frontline clinician perspectives toward antibiotic stewardship programs

**DOI:** 10.1017/ice.2023.35

**Published:** 2023-12

**Authors:** Maria Tjilos, Mari-Lynn Drainoni, Shana A. B. Burrowes, Jorie M. Butler, Laura J. Damschroder, Matthew Bidwell Goetz, Karl Madaras-Kelly, Caitlin M. Reardon, Matthew H. Samore, Jincheng Shen, Edward A. Stenehjem, Yue Zhang, Tamar F. Barlam

**Affiliations:** 1 Department of Community Health Sciences, School of Public Health, Boston University, Boston Massachusetts; 2 Section of Infectious Diseases, Department of Medicine, Chobanian and Avedisian School of Medicine, Boston University, Boston, Massachusetts; 3 Department of Health Law, Policy and Management, School of Public Health, Boston University, Boston, Massachusetts; 4 Evans Center for Implementation and Improvement Sciences, Department of Medicine, Boston University Chobanian and Avedisian School of Medicine, Boston University, Boston, Massachusetts; 5 Division of Geriatrics, Department of Internal Medicine, University of Utah, Salt Lake City, Utah; 6 Geriatric Education and Clinical Center and IDEAS Center of Innovation, Veterans’ Affairs (VA) Salt Lake City Health Care System, Salt Lake City, Utah; 7 VA Center for Clinical Management Research, Department of Veterans’ Affairs, Ann Arbor, Michigan; 8 VA Greater Los Angeles Healthcare System, Los Angeles, California; 9 David Geffen School of Medicine, UCLA, Los Angeles, California; 10 Boise VA Medical Center, Boise, Idaho; 11 College of Pharmacy, Idaho State University, Meridian Idaho; 12 IDEAS Center of Innovation, VA Salt Lake City Health Care System, Salt Lake City, Utah; 13 Divison of Epidemiology, Department of Internal Medicine, School of Medicine, University of Utah, Salt Lake City, Utah; 14 Department of Population Health Sciences, University of Utah, Salt Lake City, Utah; 15 Division of Infectious Diseases and Epidemiology, Intermountain Healthcare, Salt Lake City, Utah

## Abstract

**Objective:**

To examine the perspectives of caregivers that are not part of the antibiotic stewardship program (ASP) leadership team (eg, physicians, nurses, and clinical pharmacists), but who interact with ASPs in their role as frontline healthcare workers.

**Design:**

Qualitative semistructured interviews.

**Setting:**

The study was conducted in 2 large national healthcare systems including 7 hospitals in the Veterans’ Health Administration and 4 hospitals in Intermountain Healthcare.

**Participants:**

We interviewed 157 participants. The current analysis includes 123 nonsteward clinicians: 47 physicians, 26 pharmacists, 29 nurses, and 21 hospital leaders.

**Methods:**

Interviewers utilized a semistructured interview guide based on the Consolidated Framework for Implementation Research (CFIR), which was tailored to the participant’s role in the hospital as it related to ASPs. Qualitative analysis was conducted using a codebook based on the CFIR.

**Results:**

We identified 4 primary perspectives regarding ASPs. (1) Non-ASP pharmacists considered antibiotic stewardship activities to be a high priority despite the added burden to work duties: (2) Nurses acknowledged limited understanding of ASP activities or involvement with these programs; (3) Physicians criticized ASPs for their restrictions on clinical autonomy and questioned the ability of antibiotic stewards to make recommendations without the full clinical picture; And (4) hospital leaders expressed support for ASPs and recognized the unique challenges faced by non-ASP clinical staff.

**Conclusion:**

Further understanding these differing perspectives of ASP implementation will inform possible ways to improve ASP implementation across clinical roles.

The public health crisis of infections caused by antibiotic-resistant bacteria is largely driven by antibiotic use, much of which is inappropriate.^1^ Antibiotic stewardship programs (ASPs), which use evidence-based strategies to improve prescribing, are recommended by national public health authorities and professional organizations and are required for participation in Medicare and Medicaid.^2–4^ Antibiotic stewardship is defined as efforts to “… measure and improve how antibiotics are prescribed by clinicians and used by patients.”^2^ ASP teams are typically composed of a physician and a pharmacist who use their expertise to guide other clinical staff (eg, physicians, pharmacists and nurses) to appropriately prescribe and administer antibiotics.^2^

Successful implementation of ASPs is multifaceted and facilitated by nonstewardship physician engagement^5,6^ and clearly defined financial support^7,8^ with dedicated effort for antibiotic stewardship activities.^8,9^ In prior work, our team explored perspectives of physician and pharmacist stewards regarding successful ASP implementation.^10^ In this study, we explored the other side of the equation, the perspectives of non-ASP clinicians such as physicians, nurses, and clinical pharmacists who interacted with ASPs. As frontline healthcare workers their perspectives can be used to inform possible ways to improve ASPs.

## Methods

### Sample and recruitment

This qualitative analysis is part of a larger mixed-methods research study examining the implementation of inpatient ASPs across 2 large healthcare systems: the Veterans’ Health Administration (VA) and Intermountain Healthcare (IHC). All healthcare systems in this study employed restrictive antibiotic prescribing and audit-and-feedback strategies to varying degrees. For the larger study, we first distributed a survey based on the Consolidated Framework for Implementation Research (CFIR)^11^ to 152 physician stewards and 177 pharmacist stewards across 154 hospitals. We received survey responses from at least 1 steward at 126 hospitals (response rate, 81.8%). The survey methods have been previously described elsewhere.^12^

Using the CFIR-based survey results and prior work from the Healthcare Analysis and Information group (HAIG) survey conducted within the VA,^13^ our team used clustering analysis on the structural components of the survey to create a typology that reflected 2 different components of ASPs within hospitals: enthusiasm and implementation. The survey questions in the CFIR-based and HAIG surveys were used in the clustering analysis.^14,15^ We carefully examined the characteristics of each resulting cluster and labeled the clusters from the CFIR survey as the level of ASP enthusiasm and those from the HAIG survey as the level of ASP implementation. Level of antibiotic use was not included in creating the typology. The final typology included 4 categories: high enthusiasm–high implementation, high enthusiasm–low implementation, low enthusiasm–low implementation, low enthusiasm–high implementation. All analyses were performed using R statistical software version 4.1.3 (R Foundation for Statistical Computing, Vienna, Austria).

To examine how stewardship functioned across typologies, we conducted site visits across the 4 categories.^16^ We initially selected 4 hospitals in each typology and contacted stewards at those hospitals to determine their interest in participating. As hospitals declined or did not respond, we contacted other hospitals randomly assigned from the same categories. Ultimately, 30 hospitals across the 4 categories were contacted; of these, 14 hospitals were scheduled for site visits. Study investigators who performed the interviews were aware of the hospital typology but were blinded to the hospital’s level of antibiotic use. Hospitals were blinded to their level of antibiotic use and typology category.

We conducted 11 hospital site visits between August 2019 and March 2020; the remaining 3 site visits were cancelled due to COVID-19–related travel restrictions. There were 4 site visits in the high enthusiasm–low implementation category, 3 site visits in the high enthusiasm–high implementation category, 3 site visits in the low enthusiasm–low implementation category, and 1 site visit in the low enthusiasm–high implementation category. We worked with stewards at each site to coordinate the site visit and identify individuals within their hospital across disciplines who we could interview to gain their perspectives.

### Data collection and analysis

Semistructured qualitative interview guides were created to direct data collection during site visits. The interview guides were grounded in domains of the CFIR: intervention characteristics, outer setting, inner setting, characteristics of individuals, and process.^11^ The interview guides were further tailored to the participant’s role in the hospital as it related to ASPs. For example, interview guides for physicians were designed to understand their relationship with and perceptions of the ASP as well as any limitations the ASP may pose. Interview guides can be found in Appendix 1 (online).

At each site visit, we interviewed pharmacist and physician stewards, hospital leaders, physicians who interacted with the ASP team, clinical pharmacists, floor nurses, nurses from the hospital epidemiology and infection control teams, and microbiology laboratory staff. Interview participants varied across hospitals depending on the structure of the hospital and ASP. We conducted individual interviews and small group interviews. Individual interviews were conducted with most physician and pharmacist stewards, and group interviews were typically conducted with non-ASP clinicians (eg, nurses, physicians, pharmacists). All interview participants completed a brief demographic survey. At least 2 investigators trained in qualitative interviewing conducted all interviews. Participants provided verbal consent to participate in the study and interviews were audio-recorded with consent. The Boston University Medical Campus and Boston Medical Center Institutional Review Board approved all study protocols and granted a waiver of documentation of consent.

Interviews were transcribed verbatim by a professional transcription company; all identifying information except hospital site and role of interviewee was removed from transcripts. The analysis employed a combination of inductive and deductive methods. A preliminary codebook was developed using the constructs from the CFIR. Also, 4 members of the study team applied this CFIR-based codebook to initial transcripts independently and used emergent coding to inductively identify themes not captured by the CFIR constructs to further refine the codebook. After consensus was reached, 2 team members used the qualitative software program NVivo 12 for final coding and data analysis.

## Results

### Characteristics of study participants

We conducted interviews at 7 VA sites and 4 IHC sites. We interviewed 157 participants: 30 individually and 127 in small groups of 2–8 persons. In the current analysis, we focused on the nonsteward clinicians and included 123 participants. Among nonstewards, we interviewed 47 physicians: 21 hospitalists, 13 surgeons, 5 intensivists, 4 emergency medicine physicians, 2 infectious disease physicians, and 2 physicians who did not list a specialty. We also interviewed 26 clinical pharmacists, 29 nurses (including 3 infection prevention nurses), and 21 members of hospital leadership. Most individuals interviewed were female and had been at their institution for a median of 7 (±8) years (Table 1).

**Table 1.** Demographic Description of Non-Steward Interview Participants

Variables	Total Sample, No. (%)	Leadership, No. (%)	Nonsteward Providers, No (%)
Interview participants	123	21 (17.07)	102 (82.92)
**Sex**			
Male	58 (47.15)	12 (57.14)	46 (45.10)
Female	65 (52.85)	9 (42.86)	56 (54.90)
**Provider type**			
Pharmacist	36 (29.27)	10 (47.62)	26 (25.49)
Physician	56 (45.53)	9 (42.86)	47 (46.09)
Hospitalists	25 (44.64)	4 (44.44)	21 (44.68)
Surgeons	14 (25.00)	1 (11.11)	13 (27.7)
Intensivists	5 (8.93)	0 (0.00)	5 (10.64)
Emergency medicine	6 (10.71)	2 (22.22)	4 (8.51%)
Infectious disease	3 (5.36)	1 (11.11)	2 (4.26)
Other/Unspecified	3 (5.36)	1 (11.11)	2 (4.26)
Nurse	31 (25.20)	2 (9.52)	29 (28.43)
Years at facility, median ±IQR^a^	7±8	12±13	6±6.5
**Facility type**			
Intermountain Hospital	60 (38.22)	5 (23.81)	45 (41.28)
VA	97 (61.78)	16 (76.19)	64 (58.72)
**Geographic region**			
Northeast	10 (6.37)	4 (19.05)	4 (3.67)
Midwest	30 (19.11)	5 (23.81)	21 (19.27)
West	91 (57.96)	10 (47.62)	66 (60.55)
South	26 (16.56)	2 (9.52)	18 16.51)

Note. IQR, interquartile range; VA, Veteran’s Affairs medical center.

a Missing data for 1 participant.

### Overview of clinical perspectives

Overall, clinical staff who were not direct members of the stewardship team expressed general acceptance of ASPs, believed in their benefit for patient care, and thought ASPs were important for controlling antibiotic resistance. We identified 3 primary perspectives regarding ASPs: (1) Non-ASP pharmacists consider antimicrobial stewardship activities to be a high priority despite the added burden to work duties; (2) Nurses acknowledge limited understanding of ASP activities and involvement with these programs; (3) Physicians criticize ASPs for their perceived restrictions on clinical autonomy and question the ability of antibiotic stewards to make recommendations without the full clinical picture; And (4) hospital leaders express support for ASPs and recognize the unique challenges faced by non-ASP clinical staff. These perspectives did not vary by typology category. These themes are described below with accompanying illustrative quotes from the interviews represented in Table 2. Additional quotes of interest are provided in Appendix 2 (online).

**Table 2.** Overview of Illustrative Quotes

1. Non-ASP pharmacist consider antimicrobial stewardship activities to be a high priority despite the added burden to work duties	a. *“… [Antibiotic stewardship has] been really beneficial in managing patient infections… more appropriately, and using antibiotics more judiciously.” (#082-Pharmacist, Site 3, VA)*
	b. *“… Now that we’re putting so much emphasis on antimicrobial stewardship that’s been putting a higher strain on the pharmacists that are on the floors… And so we’re taking away from other activities that maybe we should still be doing, but we can’t.” (#085-Pharmacist, Site 4, IHC)*
	c. *“… It’s me just doing [antibiotic stewardship] things during my day-to-day, when I’m here doing all my other work. So it’s harder to not feel like you have the time to just focus on that… it would be nice to feel like I had time to directly spend on that…” (#088-Pharmacist, Site 6, IHC)*
2. Nurses acknowledge limited understanding of ASP activities or involvement with these programs	d. *“I don’t know how to explain to the patients why antibiotics are changing… Yeah, the education would be nice.” (#52-Nurse, Site 2, IHC)*
	e. “*… I don’t know how many people have ever seen the Pharmacist Steward, or know who [they are]… I don’t know whether the program is known …” (#67-Nurse, Site 8, VA)*
	f. “*I’m not clear what you mean by stewardship.” (#056-Nurse, Site 3, VA)*
	g. *“… [We are] trying to decrease the incidence of infections that would require antibiotics [such as] catheter-associated urinary tract infections… if [our patients] do have an indwelling catheter, then we’re getting them out as soon as possible.” (#048-Nurse, Site 1, VA).*
3. Physicians criticize ASPs for their perceived restrictions on clinical autonomy and question the ability of antibiotic stewards to make recommendations without the full clinical picture	h. *“… In the ER we go broad, because… We don’t know what the source is half the time, we don’t know what the bug is, we don’t have the luxury of what previous cultures show…” (#028-Emergency Medicine Physician, Site 6, IHC)*
	i. *“In terms of the potential issues on the side of the clinical providers. The most common one that I hear is, for lack of a better word, anxiety, about either stopping or lowering the duration of antibiotic treatment or moving to more narrow spectrum onto antimicrobials in somebody who is particularly sick.” (#010-Critical Care Physician, Site 3, VA)*
	j. *“You have to acknowledge that each patient is a unique case and what I don’t like is when somebody is sitting in a computer, right… without knowing the context and just deny an antibiotic because of a recipe.” (#030-Critical Care Physician, Site 7, VA)*
	k. *“… For all intents and purposes, I am aware that there’s been stewardship because of … the reins that pharmacy holds here.” (#038-Surgeon, Site 8, VA)*
	l. *“… I think it’s also important for physicians to have the autonomy to exercise [prescribing decisions without waiting] for an [infectious disease] doctor to approve a particular antibiotic…” (#037-Surgeon, Site 8, VA)*
	m. *“… If we feel that maybe physically and clinically the patient needed something different… we do get pressured a little bit more to stick with specific guidelines and that might be a problem.” (#044, Hospitalist, VA)*
4. Hospital leaders express support for ASPs and recognize the unique challenges faced by non-ASP clinical staff	n. *“… I think it’s a very important thing in terms of quality, cost, stewardship, the patient experience.” (#117-Hospital Leadership, Site 9, VA)*
	o. *“I think we very, very quickly saw the benefit of [AS] for our patients… once we implemented this and really formalized it, you saw how much more you were capturing… Really the before and after, is just the number of patients that we’re being able to impact. Our physician partners have been amazing as far as, I have not ever heard any pushback.” (#110-Hospital Leadership, Site 5, IHC)*
	p. *“…Nursing, a lot of times doesn’t know the formal side of stewardship, but they’re with the patient, they’re giving the meds, they definitely have a role.” (#110-Nursing Leadership, Site, 5, IHC)*
	q. *“…We’re not immune to that common mindset among physicians, which is, ‘well I trained at such and such clinic or such and such hospital and we learned this there and that’s why I do this.’” (#112-Hospital Leadership, Site 6, IHC)*

#### Non-ASP pharmacists considered antimicrobial stewardship activities to be a high priority despite the added burden to work duties

Non-ASP pharmacists are often asked to carry out antimicrobial stewardship activities. Pharmacists considered ASPs a priority and saw their benefit to improve patient care and control antibiotic use (Table 2, quote 1a). Yet, pharmacists also stated that antimicrobial stewardship interventions take time away from other responsibilities.

Pharmacists identified a variety of activities that suffered due to the prioritization of antimicrobial stewardship activities such as completing anticoagulation reports, performing renal dosing calculations, and taking detailed medication histories (Table 2, quote 1b). The sentiment of being stretched thin was shared by other pharmacists, who expressed that adequate staffing or dedicated time for antimicrobial stewardship activities would help reduce their added stress (Table 2, quote 1c).

#### Nurses acknowledged limited understanding of ASP activities or involvement with these programs

Although nurses felt they have an important role to play in ASPs, they also recognized the need for increased education in antibiotic prescribing principles and antibiotic stewardship programs (Table 2, quotes 2d and 2e). In response to questions about their involvement with or knowledge of antimicrobial stewardship, nurses often responded by describing infection control or prevention initiatives (Table 2, quotes 2f and 2g).

#### Physicians criticized ASPs for their perceived restrictions on clinical autonomy and questioned the ability of antibiotic stewards to make recommendations without the full clinical picture

Although physicians agreed in principle that antibiotic stewardship is important, they had concerns about its implementation. For example, emergency medicine and critical care physicians raised concerns about the ability of ASP teams to make antibiotic recommendations without a full clinical picture of a patient. One emergency department physician stated that while ASP support is appreciated, the environment of the emergency department may make antimicrobial stewardship recommendations difficult to implement (Table 2, quote 3h).

Critical care physicians also expressed anxiety related to ASPs’ lack of a full clinical picture (Table 2, quote 2i). Furthermore, another critical care physician felt strongly that the individuality of each patient in a critical care setting is not compatible with protocol-driven ASP strategies, which limit clinical autonomy (Table 2, quote 2j).

Despite acknowledging the benefit of ASPs, physicians often expressed that systems in place to support ASPs could be restrictive to their clinical autonomy and posed limitations to clinical decision making. An ASP’s perceived restriction of clinical autonomy was a frequent critique by physicians, particularly surgeons (Table 2, quote 3k). Another surgeon emphasized the importance of clinician autonomy in prescribing decisions (Table 2, quote 3l). One hospitalist also acknowledged that ASPs had the potential to limit clinical judgement and disliked restrictions (Table 2, quote 3m).

#### Hospital leaders expressed support for ASPs and recognized the unique challenges faced by non-ASP clinical staff

In general, members of hospital leadership spoke of ASPs as a priority for their institution and highlighted that ASPs were beneficial for achieving positive hospital, physician, and patient outcomes (Table 2, quotes 4n and 4o). In recognizing the benefits of ASPs, hospital leaders were aware of the challenges that non-ASP nurses, pharmacists, and physicians face when interacting with ASPs, such as increased workload burden and perceived clinician autonomy (Table 2, quotes 4p and 4q).

## Discussion

Our qualitative analysis explored multidisciplinary clinical perspectives regarding ASPs. Non-ASP clinicians believed in the benefits of ASPs but expressed some unease about their implementation and how they impact daily workflow. Pharmacists expressed concern that ASP activities added additional tasks and took priority at the expense of other responsibilities. Nurses acknowledged limited understanding of ASP activities or involvement in these programs, often describing infection control or prevention activities when asked about ASP initiatives. Nurses familiar with ASPs were concerned that adding ASP activities to nursing duties would increase workload. Although physicians supported ASPs in principle, they had 2 main criticisms of ASPs: (1) the ASP team may lack the full clinical picture necessary to make antibiotic recommendations and (2) ASPs place excessive restrictions on clinical decision-making and autonomy. Lastly, hospital leaders expressed support for ASPs and recognized the unique challenges faced by non-ASP clinical staff. These perspectives were observed across all hospitals regardless of category.

Clinical pharmacists who are not formal members of the stewardship team have been identified as key leaders within ASPs by both hospital stewards and leaders.^17^ Prior studies have reported that pharmacists believe that their role in ASPs is crucial for improving antibiotic use but that they lack dedicated time for antimicrobial stewardship activities.^9,18,19^ These data are consistent with our results; the strain of being everyday champions of antimicrobial stewardship and ensuring other responsibilities are met suggests a lack of resources allocated to nonstewardship pharmacists for ASP purposes. Bolstering clinical pharmacy resources to accommodate antimicrobial stewardship activities is an essential part of addressing this issue.

Our finding that nurses may desire a stronger knowledge base to engage in antimicrobial stewardship activities is reflected in the literature.^20–23^ Monsees et al^22^ showed nurses were less confident in understanding antibiotic choices and providing input due to lack of antimicrobial stewardship knowledge. To play an effective role in ASPs, nurses should undergo training to understand basic antibiotic prescribing principles and practice. Hospitals must also consider the appropriate role of nursing staff in ASP initiatives.

The interviews revealed that nonstewardship physicians have complex relationships with ASPs and that barriers to optimal engagement remain. Despite research highlighting that antimicrobial stewardship activities improve clinical outcomes,^5,24–26^ physicians’ expectations for autonomy in prescribing may conflict with ASP implementation. A hospital culture that respects clinician autonomy as a fundamental principle may devalue ASP strategies and recommendations if they are different from the provider’s preferences and typical practice.^18,27,28^ Our analysis suggests that physicians who are concerned with prescribing autonomy are less motivated to engage with ASP teams due to perceived restrictions of clinician autonomy. Although evidence points to the benefits of ASP models that engage physician champions^29,30^ and an inclusive approach to prescribing decisions,^31^ non-ASP physicians must also take accountability^32,33^ to engage and recognize the crucial evidence base ASP teams provide. Although physician attitudes may present barriers to engagement in ASPs, prescribing autonomy that conflicts with evidence-based medicine should not be acceptable. This finding is consistent with the finding of Jenkins and Tamma^33^ that underscores the importance of shifting the ASP model to one that places a greater responsibility on physicians to utilize evidence-based prescribing practices.

Furthermore, physicians can mistrust or be skeptical of input from antibiotic stewards who are not members of the direct patient care team,^10,27,34,35^ as has been reported in studies of hospitalists,^29,30,36^ intensivists,^37,38^ and surgeons.^39,40^ In our analysis, physicians’ desire for autonomy was accompanied by a perception that ASP teams lack the full clinical picture to provide prescribing advice.

Our results indicate that hospital leaders are supportive of ASPs and are aware of the unique challenges faced by frontline healthcare providers in relationship to ASPs. They are uniquely positioned to understand the perspectives of frontline healthcare providers toward ASP engagement and the need to balance their other clinical responsibilities. For example, both pharmacists and nurses expressed hesitation for more involvement due to the possibility of added patient care responsibilities. In efforts to improve ASP implementation, it is crucial to weigh the advantages of an interdisciplinary approach to ASPs against the disadvantages of added healthcare provider burnout, which may compromise positive health outcomes and healthcare workforce morale.

These non-ASP clinician perspectives were observed across all hospitals regardless of where the hospital fit into the typology. This finding may be because typology categories were created based on surveys completed by stewards and therefore were not representative of non-ASP clinician perspectives. Interviewees expressed personal perspectives on ASP implementation, and whether their perspectives are related to ASP outcomes remains unclear because we did not collect outcome data. Further research is necessary to better elucidate the relationship between steward attitudes toward ASP implementation, non-ASP perspectives of ASP implementation, and ASP outcomes.

This study had several limitations. First, stewards at each site selected individuals from their hospital to be interviewed by our team. Stewards may have chosen individuals they thought would express favorable views toward stewardship; thus, our participants’ views may not be fully reflective of true attitudes of the full team toward ASPs. Second, we only interviewed individuals at 2 hospital systems; the VA is a federally run hospital system whereas IHC is a hospital system in Utah. As with any qualitative study, our results are not meant to be generalizable because they represent the perspectives of our specific study sample. However, our study provides important findings that can inform ASP implementation and resources.

In summary, non-ASP clinical pharmacists, nurses, and physicians demonstrated differing perspectives of ASPs. Based on our results, we recommend that ASPs and physicians meet each other where they are for successful ASP implementation. The creation of meaningful partnerships between ASPs and non-ASP pharmacists and nurses may better engage them by establishing clear roles for them in ASP processes, training, and implementation. Hospital leaders are critical stakeholders with a deep understanding of the goals of ASPs and the distinct perspectives of frontline healthcare providers. They can provide the necessary financial and human resources to bolster ASP implementation while fostering a supportive organizational culture. Our results and recommendations further strengthen existing literature that highlights similar facilitators of ASPs such as collaborative, respectful relationships between ASPs and frontline clinicians, establishment of clear nursing roles, and working together as a team, among others.^10,18–20^

Additionally, while physician autonomy is important, evidence-based practice is critical for successful outcomes. Rather than viewing themselves as unwilling recipients of ASPs, physicians must view themselves as accountable leaders within ASPs and must see ASPs as a critical resource to enhance their medical practice. In highlighting the perceptions and needs of non-ASP pharmacists, nurses, and physicians, we underline important considerations for future engagement of these clinicians to increase judicious use of antibiotics and elevate patient care.

## Supplementary material

For supplementary material accompanying this paper visit https://doi.org/10.1017/ice.2023.35. click here to view supplementary material
