# Supplementary material for: A qualitative evaluation of frontline clinician perspectives toward antibiotic stewardship programs
Source: Infect Control Hosp Epidemiol. 2023 Mar 29;44(12):1995–2001. doi: 10.1017/ice.2023.35 (PMC10755145; doi:10.1017/ice.2023.35)
Supplement: Supplementary file 1 [file S0899823X23000351sup.zip › S0899823X23000351sup001.docx]

**Supplemental Appendix 2: Additional Illustrative Quotes**

***Quotes from non-ASP Pharmacists***

*“I think [stewardship is] essential. I think we really need to do what we're doing for the safety of our patients and for the effectiveness of antibiotics in general…”(#085-Pharmacist, Site 2, IHC)*

“…*we try not to be pushy about [antibiotic stewardship]. We find that it's easier to just say, ‘I would suggest this,’ or letting them ask us for questions rather than hammering it home, because it kind of creates animosity when you're saying, ‘You did this wrong. I totally don't agree with what you're doing.’ And by compromising at times… they've been a lot more agreeable to letting us make recommendations on their cases.”(#077-Pharmacist, Pharmacist, VA)*

“*I think [physicians are] very receptive. I think we have good relationships with all of our physicians just because we are such a small facility. So they are more than happy to either contact the Infectious Disease doctors when we recommend it or if we make a recommendation, they usually trust us enough to take that recommendation as well for changing therapy. So I think it's a good relationship.” (#087-Pharmacist, Site 6, IHC)*

***Quotes from non-ASP Nursing***

*“We've been trying to recruit nursing staff to be a member of the Stewardship team, but no takers yet… possibly [because people are overworked].” (01_Medical Surgery Nursing Manager, VA)*

*“Yeah, the education would be nice. I don't think I want that responsibility, not having enough training in the antibiotics and sensitivity. I don't think I want that responsibility with that patient's care if I chose the wrong one.” (#052-Nurse, Site 2, IHC)*

***Quotes from Hospital Leadership***

*“[my role is] to make sure that the resources are there for the program. I will say I'm not really involved in the day in, day out running of the program, but I have regular meetings with all of my clinical service chiefs and right now we are in the middle of our planning for this coming fiscal year on what resources services are going to need to fulfill mission and they are not shy about letting us know what they feel they need.” (#108-Hospital Leadership, Site 3, VA)*

*“this is a very important thing. I mean antibiotics are obviously over prescribed, we have issues every day with the antibiotic, the development of antibiotic… resistance…” (#117-Hospital Leadership, Site 9, VA)*

*“…we're not immune to that common mindset among physicians, which is, "Well I trained at such and such clinic or such and such hospital and we learned this there and that's why I do this." (#112-Hospital Leadership, Site 6, IHC)*

***Quotes from non-ASP Physicians***

*“I think all the pharmacists in our facility are great, but I really love ours because they're very very committed and keen and keep us [up to date on] what's the latest, and here's what we're doing now, and we're not doing that anymore. In this population, here's our antibiogram. This is what we need to be using, and those types of things.” (#047-Critical Care Physician, Site 11, IHC)*

*“I don't think of [ASP] as a separate team. They're part of the team when we round, they're helping make patient care decisions, recommendations, [they’re] incorporated into the way we practice… [collaboration] makes [stewardship] nice.” (#005-Surgeon, Site 1, VA)*

*“It depends on how ill the patient is acutely. Sometimes you need to prescribe very broad spectrum antibiotics for someone with severe sepsis… it's pretty easy to pick a broad coverage. But when you're picking targeted coverage, sometimes it can be a little trickier especially if the patient has complex allergies or has been on antibiotics recently, then it's case by case.” (#026-Emergency Medicine Physician_Site 2, IHC)*
