# Supplementary material for: A qualitative evaluation of frontline clinician perspectives toward antibiotic stewardship programs
Source: Infect Control Hosp Epidemiol. 2023 Mar 29;44(12):1995–2001. doi: 10.1017/ice.2023.35 (PMC10755145; doi:10.1017/ice.2023.35)
Supplement: Supplementary file 1 [file S0899823X23000351sup.zip › S0899823X23000351sup002.docx]

**Focus Group Guide – Inpatient Clinical Pharmacists**

1. **What impact does the stewardship program have on your work?**

Probes: Does stewardship pose a big burden? When implementing orders on the floor, do you get pushback related to stewardship?

1. **What do you think about antibiotic stewardship in general?**

Probes: Is it important? Do you think folks at the hospital think it’s important?

1. **Is there anything else you want to share about your experience with the antibiotic stewardship program here that we should know?**

**Focus Group Guide – Inpatient Nursing Staff**

1. **What impact does the stewardship program have on your work?**

Probes: Does stewardship pose a big burden?

1. **What do you think about antibiotic stewardship in general?**

Probes: Is it important? Do you think folks at the hospital think it’s important?

1. **Is there anything else you want to share about your experience with the antibiotic stewardship program here that we should know?**

**Focus Group Guide – Medical Providers**

1. **In what sorts of situations do you interact with your hospital stewardships team?**
2. **Who is part of the stewardship team here? Who do you interact with the most? When do you tend to interact with them?**
   1. Probes: In what situations do you prefer their expertise? In what situations do you rely on your training? How much do you interact with stewards?
3. **What do you think about antibiotic stewardship?**
   1. Probes: Is it important? Is it limiting? How important is it that your prescribing is in line with other providers at your hospital? For those who were here before stewardship program was implemented, what changes have you seen?
4. **How much education have you received about the stewardship program?**
   1. Probes: Has it been useful? How are you told about changes or updates to the stewardship program?
5. **[If applicable] What is your perception of the tele-stewardship program?**
   1. Probes: Benefits of the model? Drawbacks?
6. **Is there anything else you want to share about your experience with the antibiotic stewardship program here that we should know?**

**Group Interview Guide – Hospital Leadership**

1. **When did you start planning an antibiotic stewardship program to improve appropriate antibiotic use? What were the factors that led up to starting a stewardship program?**
2. **How involved are you in stewardship activities? Can you describe specifically what you do?**
3. **What are your priorities related to stewardship? How, if at all, has prioritizing stewardship changed your hospital culture?**

Probes: How do you view the relative importance of antibiotic stewardship *compared to other hospital and organizational initiatives*?

1. **What resources have been allocated to support and sustain the antibiotic stewardship program?**
2. **What changes or adaptations to the antibiotic stewardship program would you like to see?**

Probes: Stewardship team composition; site visibility; data feedback

1. **Is there anything else you want to share about your experience with the antibiotic stewardship program here that we should know?**
